# Sick building syndrome and associated risk factors among the population of Gondar town, northwest Ethiopia

**DOI:** 10.1186/s12199-018-0745-9

**Published:** 2018-10-27

**Authors:** Haileab Belachew, Yibeltal Assefa, Gebisa Guyasa, Jember Azanaw, Tsegaye Adane, Henok Dagne, Zemichael Gizaw

**Affiliations:** Department of Environmental and Occupational Health and Safety, Institute of Public Health, College of Medicine and Health Sciences, University of Gondar, Gondar, Ethiopia

**Keywords:** Sick building syndromes, Residential buildings, Building-related symptoms, Gondar town

## Abstract

**Background:**

Sick building syndrome (SBS) consists of a group of mucosal, skin, and general symptoms temporally related to residential and office buildings of unclear causes. These symptoms are common in the general population. However, SBS symptoms and their contributing factors are poorly understood, and the community associates it with bad sprits. This community-based cross-sectional study was, therefore, conducted to assess the prevalence and associated factors of SBS in Gondar town.

**Methods:**

A community-based cross-sectional study was conducted from March to April 2017. A total of 3405 study subjects were included using multistage and systematic random sampling techniques. A structured questionnaire and observational checklists were used to collect data. SBS was assessed by 24 building-related symptoms and confirmed by five SBS confirmation criteria. Multivariable binary logistic regression analysis was used to identify factors associated with SBS on the basis of adjusted odds ratio (AOR) with 95% confidence interval (CI) and *p* < 0.05. The Hosmer and Lemeshow goodness of fit test was used to check model fitness, and variance inflation factor (VIF) was also used to test interactions between variables.

**Results:**

The prevalence of SBS in Gondar town was 21.7% (95% CI = 20.3–23.0%). Of this, the mucosal symptoms account for 64%, the general symptoms account for 54%, and the skin symptoms account for 10%. From study participants who reported SBS symptoms, 44% had more than one symptom. Headache (15.7%), asthma (8.3%), rhinitis (8.0%), and dizziness (7.5%) were the commonest reported symptoms. SBS was significantly associated with fungal growth in the building [AOR = 1.25, 95% CI = (1.05, 1.49)], unclean building [AOR = 1.26, 95% CI = (1.03, 1.55)], houses with no functional windows [AOR = 1.35, 95% CI = (1.12, 1.63)], houses with no fan [AOR = 1.90, 95% CI = (1.22, 2.96)], utilization of charcoal as a cooking energy source [AOR = 1.40, 95% CI = (1.02, 1.91)], cooking inside the living quarters [AOR = 1.31, 95% CI = (1.09, 1.58)], and incensing and joss stick use [AOR = 1.48, 95% CI = (1.23, 1.77)].

**Conclusion:**

The prevalence of SBS in Gondar town was high, and significant proportion of the population had more than one SBS symptom. Headache, asthma, rhinitis, and dizziness were the commonest reported SBS symptoms. Fungal growth, cleanliness of the building, availability of functional windows, availability of fan in the living quarters, using charcoal as a cooking energy source, cooking inside the quarters, and incensing habit or joss stick use were identified as factors associated with SBS. Improving the sanitation of the living environment and housekeeping practices of the occupants is useful to minimize the prevalence of SBS.

## Background

Housing is one of the basic needs of a human being and fundamental for wellbeing [1, 2]. People spend more than 90% of their time indoors [3]. Housing increasingly becomes a major public health concern. For many years, the housing environment has been acknowledged as one of the main settings that affect human health. Indoor air quality, home safety, noise, humidity and mold growth, indoor temperatures, asbestos, lead, radon, volatile organic compounds (VOC), lack of hygiene and sanitation equipment, and crowding are some of the most relevant possible health threats in dwellings [4–6].

Physical, mental, and social health is affected by the living conditions. The quality of housing conditions plays a decisive role in the health status of the residents. Many health problems are either directly or indirectly related to the building itself, because of the construction materials that were used and the equipment installed or the size or design of the individual dwellings [4, 7]. These building-related health problems are categorized in building-related diseases and sick building syndrome (SBS) [8]. SBS consists of a group of mucosal, skin, and general symptoms that are temporally related to residential and office buildings. SBS comprises a group of symptoms of unclear causes divided into mucous membrane symptoms related to the eyes, nose, throat, and dry skin, together with what are often called general symptoms of headache and lethargy [7–9]**.**

SBS can be influenced by a variety of factors, like building-related factors (air-conditioned building, fresh air ventilation rates, indoor temperature, poor building service maintenance and cleaning, relative humidity) [7, 10–13], environmental factors and pollutants [VOCs (formaldehyde, solvents, etc.), carbon monoxide (stoves, heaters, and furnaces), dust and fibers (asbestosis, fiberglass, dirt), bio-aerosols (bacteria, molds, viruses, pollen, dust, mites, animal danders, animal excreta), trapped outdoor pollutants (vehicle or industrial exhausts), physical factors (lighting, vibration, noise, temperature, crowding, photo duplication)] [14–17], and personal factors (gender, history of being allergic, job dissatisfaction, cigarette smoke, increased use of computers) [18–22].

SBS symptoms are common in the general population. However, SBS symptoms and their contributing factors are poorly understood, especially in developing countries including Ethiopia. The community associates SBS with bad spirit. This community-based cross-sectional study was, therefore, conducted to assess prevalence and contributing factors of SBS in Gondar town.

## Methods

### Study design and description of study settings

A community-based cross-sectional study was conducted from March to April 2017 in Gondar town. Gondar town is located in the northern part of Ethiopia in Amhara National Regional State, North Gondar Zone, at a distance of 747 km from Addis Ababa and 170 km from Bahirdar at 12° 45′ north latitude and 37° 45′ east longitudes. Gondar was founded in 1643. Based on the 2016 population estimate, Gondar had a total population of 621,168 with 3200/km^2^ [23].

### Sample size determination

Single-population proportion formula [24] was used to determine the sample size (*n*) with the following assumptions: *p* (prevalence of SBS = 50%, hence there are no other similar studies in Ethiopia), 95% confidence interval (standard normal probability = 1.96); 3% margin of error or maximum error to commit, *z* = the standard normal tabulated value, and *α* = level of significance.

$$ n=\frac{{\left({z}_{\raisebox{1ex}{$\alpha $}\!\left/ \!\raisebox{-1ex}{$2$}\right.}\right)}^2p\left(1-p\right)}{d^2}=\frac{(1.96)^20.5\left(1-0.5\right)}{0.03^2}=1068 $$

We used a design effect of 3 based on the recommendation of the Population Services International research tool kit [25] and 7% non-response rate; the final sample became 3429.

### Sampling procedures

Multistage sampling technique was used to select study participants. Seven kebeles (the lowest administrative unit in Ethiopia) were selected from a total of 22 kebeles using simple random sampling technique. Residential buildings found in the selected kebeles were chosen using systematic random sampling technique in every seven interval. The first residential building was selected from seven houses by lottery method.

### Data collection procedures

A structured questionnaire and observational checklist were used to collect data. The questionnaire was pre-tested out of the study area in a community which had similar characteristics prior to the actual data collection. Twelve graduating class environmental health students were involved in the data collection process. Training was given for the data collectors and supervisors. The data collectors visited all systematically selected households and interviewed all household members. For under-5-year-old household members, data collectors interviewed mothers or caregivers. Data collectors asked the study subjects to recall the presence of 24 SBS symptoms and related information in 3 months prior to the survey. Collectors also observed the housing and living environment condition. The overall interview process was supervised by supervisors. The collected data were checked and corrected by the data collectors immediately after finalizing the questionnaire before they left the house. Supervisors daily checked the completeness, quality, and consistency of information collected.

### Measurement of study variables

SBS, the primary outcome variable of this study, was defined as the presence of at least one symptom associated with housing condition in the last 3 months prior to the survey. SBS was assessed by asking have you had any (or more) of the following symptoms during the last 3 months: (i) general symptoms including fatigue, headache, dizziness, reduced attention, hyperactivity, fever, chills, and eye strain; (ii) mucosal symptoms including rhinitis, nasal congestion, wheezing, asthma, dyspnea, severe lung disease, epistaxis, upper respiratory tract irritation, chest tightness, dry throat, cough, and eye irritation; and (iii) skin symptoms including skin rashes, dry or flushed facial skin, scaling/itching scalp or ears, and lip dryness [9, 26, 27]. Five criteria were used to confirm whether the symptoms are SBS or not. The criteria were as follows: (a) symptoms aggravate when staying at home, (b) symptoms either immediately or gradually disappear when leaving the house, (c) symptoms recur when returning home, (d) symptoms aggravate during the night, and (e) symptoms disappear when the room is ventilated or cleaned.

The wealth index of households was determined using principal component analysis (PCA). As health and demographic surveys (DHS) recommended, we used asset and service variables to determine the wealth index. Initially, the wealth index was classified into very poor, poor, moderate, rich, and very rich. But there was no significant difference between very poor and poor, and moderate, rich, and very rich. Based on this fact, the wealth index was classified into poor and rich. Residential buildings were taken as clean if the physical structures (floors, walls, ceilings, or roofs) have no any visible dirt, soot, spider’s wrap, crack, and dampness. The living compound was taken as clean if the living environment is free from wastes, vectors, and unpleasant odor. The illumination system of the living quarters was taken as adequate if the light energy is constant, free from glare, uniformly distributed to the entire room, and suitable to perform daily activities inside without strain.

### Data management and statistical analysis

Data were entered using EPI-INFO version 7 and exported into SPSS version 20 for further analysis. For most variables, data were presented by frequencies and percentages. Univariable binary logistic regression analysis was used to choose variables for the multivariable binary logistic regression analysis, variables with *p* value less than 0.05 by the univariable analysis were then analyzed by multivariable binary logistic regression for controlling the possible effect of confounders (like age, sex, family size, economic status, and education status of the family), and finally, variables which had significant association with SBS were identified on the basis of AOR with 95% CI and *p* < 0.05. The Hosmer and Lemeshow goodness of fit test was used to check model fitness. VIF was also used to test interactions between variables.

## Results

### Socio-demographic information

A total of 964 residential buildings were visited, and a total of 3405 residents in these houses were included in this study with 99.4% response rate. One thousand eight hundred thirty-eight (54%) study subjects were female. Three fourth, 2620 (76.9%), of the study subjects were aged between 15 and 64 years. Nine hundred thirty-two (27.4%) participants graduated from colleges or universities. One thousand three hundred ninety-one (40.9%) study subjects were not engaged at the time of the survey, and 1179 (34.6%) participants were self-employed. Two thousand twenty-three (59.4%) study subjects were economically poor (Table 1).

**Table 1:** Socio-demographic information of study participants in Gondar town, northwest Ethiopia, March–April 2017

Socio-demographic variables	Frequency	Percentage
Sex		
Male	1567	46.0
Female	1838	54.0
Age		
< 15 years	644	18.9
15–64 years	2620	76.9
> 64 years	141	4.1
Educational status		
Underage	171	5.0
Kindergarten	123	3.6
Cannot read and write	238	7.0
Can read and write	439	12.9
Primary education	589	17.3
Secondary education	913	26.8
College or university	932	27.4
Marital status		
Underage	629	18.5
Currently engaged	1385	40.7
Currently not engaged	1391	40.9
Occupational status		
Underage	82	2.4
Student	1072	31.5
Unemployed	220	6.5
Civil servant	609	17.9
Self employed	1179	34.6
Retired	243	7.1
Economic status		
Poor	2023	59.4
Rich	1382	40.6

### Housing condition

The majority, 2389 (70.2%), of the study subjects lived in houses constructed from wood and mud. One thousand seven hundred eighty (52.3%) study subjects reported that the floor of their house is earthen floor. Two thousand eighty-six (61.3%) study subjects said that they had only one bedroom. The majority, 3217 (94.5%), of the study participants reported that they had no fan in their house. Nearly two thirds, 2211 (64.9%), of the study subjects said that their residential building has no functional windows. One thousand seven hundred sixty-six (51.9%) study subjects lived in houses in which the illumination system was not adequate. Almost all, 3317 (97.4%), of the study participants reported that they got light from electricity. A quarter, 879 (25.8%), of the study participants had pets in the home. Fungal growth and dampness was observed among 1462 (42.9%) participants’ house. One thousand three hundred seventy-six (40.4%) study subjects reported that they recently used pesticides, paints, and solvents. One thousand eight hundred eighty-five (55.4%) participants reported that cooking inside the living quarters is a common practice. One thousand three hundred twenty-seven (39%) study subjects said that outdoor air pollutant sources (like garages, and metal and woodwork houses) were found around their home (within a 200-m radius). Incensing and utilization of a joss stick was a common habit in 1329 (39%) participants’ house. Nearly one tenth, 335 (9.8%), of the study subjects reported that at least one of the family members smokes cigarette. The houses of 1196 (35.1%) study subjects were newly constructed (within 10 years), and the houses of 1295 (38%) subjects were clean (Table 2).

**Table 2:** Housing condition of study participants in Gondar town, northwest Ethiopia, March–April 2017

Housing- and sanitation-related variables	Frequency	Percentage
Wall constructed from		
Brick or block	1016	29.8
Wood and mud	2389	70.2
Types of floor materials		
Earth floor	1780	52.3
Wood floor	347	10.2
Ceramic/tiles/brick floor	191	5.6
Cement floor	1087	31.9
Number of bedrooms		
No separate bedroom	545	16.0
1	2086	61.3
2	531	15.6
3	243	7.1
Fan is available in the quarters		
Yes	188	5.5
No	3217	94.5
Functional windows		
No	2211	64.9
Yes	1194	35.1
Illumination system of the building		
Adequate	1639	48.1
Not adequate	1766	51.9
Light sources		
Electricity	3317	97.4
Solar cell	79	2.3
Candle or kuraz	9	0.3
Pets in the home		
Yes	879	25.8
No	2526	74.2
Fungal growth is observed in the building		
Yes	1462	42.9
No	1943	57.1
Pesticides, paints, and solvents used recently		
Yes	1376	40.4
No	2029	59.6
Cooking inside		
Yes	1885	55.4
No	1520	44.6
Outdoor air pollutant sources within a 200-m radius		
Yes	1327	39.0
No	2078	61.0
Incensing habit and joss stick use		
Yes	1329	39.0
No	2076	61.0
Cigarette smoking		
Yes	335	9.8
No	3070	90.2
Building age		
New	1196	35.1
Old	2209	64.9
Cleanliness of the building		
Clean	1295	38.0
Not clean	2110	62.0

### Sanitation of the living environment

Nearly two thirds, 2192 (64.4%), of the study participants used traditional pit latrine, and three fourths, 2600 (76.4%), of study subjects got drinking water from an in-compound tap. Three thousand sixteen (88.6%), 43 (1.3%), and 1892 (55.6%) of the study participants reported that they use charcoal, kerosene, and electricity, respectively, as household energy sources. A quarter, 887 (26%), of the participants reported vector infestation in their living environment, and the living environment of 2255 (66.2%) study subjects was clean (Table 3).

**Table 3:** Sanitation practices of study participants in Gondar town, northwest Ethiopia, March–April 2017

Sanitation-related variables	Frequency	Percentage
Toilet or latrine facilities		
Flush toilet	948	27.8
Traditional pit latrine	2192	64.4
Ventilated improved pit latrine	63	1.9
No sanitation facility	202	5.9
Drinking water sources		
In-residence tap	589	17.3
In-compound tap	2600	76.4
Out-of-compound tap	216	6.3
Use charcoal as energy source		
Yes	3016	88.6
No	389	11.4
Use kerosene as energy source		
Yes	43	1.3
No	3362	98.7
Use electricity as energy source		
Yes	1892	55.6
No	1513	44.4
Infestation of vectors		
Yes	887	26.0
No	2518	74.0
Cleanliness of living compound		
Clean	2255	66.2
Not clean	1150	33.8

### Prevalence of sick building syndromes

From a total of 3405 participants included in this study, 738 participants reported one or more symptoms associated with poor housing condition. The prevalence of SBS in Gondar town was therefore found to be 21.7% (95% CI = 20.3–23.0%). Ninety (12.2%) occupants who had one or more symptoms reported that the symptoms always occurred, and 648(87.8%) occupants who had SBS said that the symptoms occurred sometimes in the last 3 months. From study participants who reported symptoms related with housing, 414 (56%) and 324 (44%) had one and more than one symptom/s, respectively. Four hundred seventy-four (64%), 401 (54%), and 72 (10%) of the reported symptoms were mucosal, general, and skin symptoms, respectively. Headache, 221 (15.7%); asthma, 116 (8.3%); rhinitis, 112 (8.0%); and dizziness, 106 (7.5%), were the commonest reported symptoms (Table 4).

**Table 4:** Symptoms related with housing as reported by study participants in Gondar town, northwest Ethiopia, March–April 2017

Symptoms	Frequency	Percentage
General symptoms		
Headache	221	15.7
Dizziness	106	7.5
Fatigue	97	6.9
Fever	79	5.6
Chills	36	2.6
Hyperactivity	34	2.4
Reduced attention	16	1.1
Eye strain	25	1.8
Mucosal symptoms		
Rhinitis	112	8.0
Nasal congestion	92	6.6
Wheezing	55	3.9
Asthma	116	8.3
Dyspnea	18	1.3
Severe lung disease	20	1.4
Epistaxis	91	6.5
Upper respiratory tract irritation	10	0.7
Chest tightness	15	1.1
Dry throat	42	3.0
Cough	60	4.3
Eye irritation	69	4.9
Skin symptoms		
Skin rashes	23	1.6
Dry or flushed facial skin	17	1.2
Scaling/ itching scalp or ears	17	1.2
Lip dryness	33	2.4

### Factors associated with sick building syndromes

Univariable binary logistic regression was used to choose variables for the final model on the basis of *p* values less than 0.05. Types of wall; fungal growth; cleanliness of the building; presence of functional windows; presence of fan in the quarters; cooking inside the quarters; charcoal use; habits of cigarette smoking; incensing habit and joss stick use; outdoor pollutant sources near the building; recent utilization of pesticides, paints, and solvents; and cleanliness of the living compound were variables selected for the final model. VIF was calculated considering one independent variable as the dependent variable turn by turn to test interactions between variables. The test result shows that VIF for all variables was below 3, threshold for collinearity diagnostics. This showed that there is no multicollinearity effect between independent variables.

Table 5 shows variables associated with SBS. SBS was statistically associated with fungal growth in the building. The probability of having SBS was 1.25 times higher among participants where fungal growth was observed in the building [AOR = 1.25, 95% CI = (1.05, 1.49)]. This study depicted that cleanliness of buildings was significantly associated with SBS. The odds of SBS was 1.26 times higher among occupants whose building is not clean compared with their counterparts [AOR = 1.26, 95% CI = (1.03, 1.55)]. The prevalence of SBS was 1.35 times higher among participants who lived in houses with no functional windows [AOR = 1.35, 95% CI = (1.12, 1.63)]. As revealed by this study, SBS was associated with utilization of fan. The probability of developing SBS was 1.90 times higher among study subjects who did not use fan [AOR = 1.90, 95% CI = (1.22, 2.96)]. This community-based study explored that SBS was associated with household cooking energy, cooking practice, and incensing habits of occupants. The odds of SBS was 1.4 times higher among occupants who used charcoal as cooking energy source [AOR = 1.40, 95% CI = (1.02, 1.91)]. Occupants who cooked inside the living quarters had more chance to develop SBS [AOR = 1.31, 95% CI = (1.09, 1.58)]. The probability to have SBS was 1.48 times higher among occupants who had a habit of incensing and using a joss stick [AOR = 1.48, 95% CI = (1.23, 1.77)].

**Table 5:** Factors associated with sick building syndromes among the population of Gondar town, northwest Ethiopia, March–April 2017

Variables	SBS		COR with 95% CI	AOR with 95% CI
	Yes	No		
Wall constructed from				
Brick or block	196	820	1	
Wood and mud	542	1847	1.23(1.02, 1.47)	1.10(0.89, 1.36)
Fungal growth is observed in the building				
Yes	362	1100	1.37(1.16, 1.62)	1.25(1.05, 1.49)*
No	376	1567	1	
Cleanliness of the building				
Clean	224	1071	1	
Not clean	514	1596	1.54(1.29, 1.83)	1.26(1.03, 1.55)*
Functional windows				
Yes	510	1701	1	
No	228	966	1.27(1.07, 1.51)	1.35(1.12, 1.63)**
Availability of fan in the quarters				
Yes	27	161	1	
No	711	2506	1.69(1.12, 2.57)	1.90(1.22, 2.96)**
Charcoal use				
Yes	684	2332	1.82(1.35, 2.46)	1.40(1.02, 1.91)*
No	54	335	1	
Cooking inside				
Yes	478	1407	1.65(1.39, 1.95)	1.31(1.09, 1.58)**
No	260	1260	1	
Family members smoke cigarette				
Yes	88	247	1.33(1.03, 1.72)	1.24(0.94, 1.63)
No	650	2420	1	
Incensing habit and joss stick use				
Yes	368	961	1.77(1.50, 2.08)	1.48(1.23, 1.77)***
No	370	1706	1	
Pollutant sources within the near distance				
Yes	312	1015	1.19(1.01, 1.41)	0.99(0.82, 1.18)
No	426	1652	1	
Pesticides, paints, and solvents used recently				
Yes	329	1047	1.25(1.06, 1.47)	1.14(0.95, 1.37)
No	409	1620	1	
Cleanliness of the compound				
Clean	444	1811	1	
Not clean	294	856	1.40(1.18, 1.66)	1.16(0.96, 1.40)

Hosmer and Lemeshow test = 0.154 showed that the model fitted well

*Statistically significant at *p* < 0.05

**Statistically significant at *p* < 0.01

***Statistically significant at *p* < 0.001

## Discussion

This study found that prevalence of SBS was 21.7% (64% mucosal symptoms, 54% general symptoms, and 10% skin symptoms). The prevalence of mucosal, general, and skin symptoms reported by this study is higher than the findings of other studies in China which reported mucosal symptoms (7.1%), general symptoms (11.4%), and skin symptoms (4.4%) [27]. Another study in China reported lower prevalence (mucosal symptoms (35.1%), general symptoms (39.4%,) and skin symptoms (43.4%)) [9] compared to the current study with exception of skin symptoms. The current prevalence is lower than that report in a study in three North European cities. The prevalence reported by the later study was 30.8% (20% mucosal, 10% general, and 8% dermal symptoms) [28]. The finding of the current study is also lower than that of a study in China that reported 74.3% mucosal symptoms, 78.7% general symptoms, and 47.5% skin symptoms [29]. The variations of prevalence among different studies may be due to differences in housing and environmental conditions. Most of the houses in the current study area were substandard. The outdoor air in the settings of the other studies is polluted by industrial emissions compared with the current setting.

This study depicted that cleanliness of residential buildings was statistically associated with SBS. The prevalence of SBS was higher among occupants who lived in unclean buildings compared with occupants who lived in clean buildings. This finding is in line with the findings of other similar studies [7, 9, 19, 30]. This may be due to the fact that unclean building surfaces including carpets accumulate dust and dirt, which are reservoirs for chemicals, allergens, and diseases causing pathogens [31, 32].

SBS was significantly associated with infestation of fungus or molds in the living building. Occupants who lived in buildings where fungal growth was observed reported SBS compared with their counterparts. The finding of this study is supported by other studies [7, 16, 33]. This is due to the fact that fungus or molds cause health problems in the mechanisms of either infection or allergy or toxin. Fungal spores are generally recognized as important causes of respiratory allergies [16, 34].

The current study explored that availability of functional windows is statistically associated with SBS. A wide range of literature also reported the effect of general ventilation on the health of the occupants [19, 35–37]. This fact can be explained that presence of functional windows as means of ventilating a building naturally helps the external fresh air to the living quarters and removes the internal exhausted air which in turn reduces the amount of contamination with chemicals or microorganisms, so that increased ventilation can be seen as an effective treatment of SBS [19, 37–39]. This study also revealed that availability of fan in the living quarters was significantly associated with SBS. The prevalence of SBS was higher among occupants who lived in houses with no fan. Other studies also reported similar findings [40, 41]. This is because fan-assisted ventilation improves the quality of the indoor air [42, 43].

This study reported that SBS was associated with household cooking energy sources, cooking practice, and incensing habits of occupants. The prevalence of SBS was higher among occupants who used charcoal as cooking energy source. Occupants who used charcoal and cooked inside the living quarters [44–46] and who used incensing and joss stick [27, 29] had more chance to develop SBS**.** This can be justified that charcoal use and incensing habits are incomplete combustion processes that can generate gracious pollutants. Generally, cooking energy sources and cooking practices are the main sources for gracious pollutants to the indoor air [47, 48].

### Limitation of the study

This research did not assess the condition of office buildings in this study though the condition of office buildings is a covariate for SBS associated with residential buildings. Moreover, we did not measure indoor air quality, thermal condition, and light intensity using instruments. However, we used standardized checklists to assess these parameters.

## Conclusion

The prevalence of SBS in Gondar town was found to be high, and a significant proportion of the population had more than one SBS symptom. Headache, asthma, rhinitis, and dizziness were the commonest reported SBS symptoms. Fungal growth, cleanliness of the building, availability of functional windows, availability of fan in the living quarters, using charcoal as cooking energy source, cooking inside the quarters, and incensing habits or joss stick use were identified as factors associated with SBS. Improving the sanitation of the living environment and housekeeping practice of the occupants is useful to minimize the prevalence of SBS.

## Abbreviations

AOR: Adjusted odds ratio
CI: Confidence interval
COR: Crude odds ratio
DHS: Health and demographic surveys
PCA: Principal component analysis
SBS: Sick building syndrome
SPSS: Statistical Package for Social Sciences
VIF: Variance inflation factor
VOCs: Volatile organic compounds
